# Gene signature and immune cell profiling by high-dimensional, single-cell analysis in COVID-19 patients, presenting Low T3 syndrome and coexistent hematological malignancies

**DOI:** 10.1186/s12967-021-02805-6

**Published:** 2021-04-01

**Authors:** Salvatore Sciacchitano, Claudia De Vitis, Michela D’Ascanio, Simonetta Giovagnoli, Chiara De Dominicis, Andrea Laghi, Paolo Anibaldi, Andrea Petrucca, Gerardo Salerno, Iolanda Santino, Rachele Amodeo, Maurizio Simmaco, Christian Napoli, Agostino Tafuri, Arianna Di Napoli, Andrea Sacconi, Valentina Salvati, Gennaro Ciliberto, Maurizio Fanciulli, Giulia Piaggio, Luisa de Latouliere, Alberto Ricci, Rita Mancini

**Affiliations:** 1 Department of Clinical and Molecular Medicine, Sapienza University, Via di Grottarossa, 1035/1039, 00189 Rome, Italy; 2 Laboratory of Biomedical Research, Niccolò Cusano University Foundation, Via Don Carlo Gnocchi, 3, 00166 Rome, Italy; 3 Division of Pneumology, Sant’Andrea Hospital, Via di Grottarossa, 1035/1039, 00189 Rome, Italy; 4 Department of Medical and Surgical Sciences and of Translational Medicine, Sapienza University, Sant’Andrea Hospital, Via di Grottarossa, 1035/1039, 00189 Rome, Italy; 5 Health Managment Director, Sant’Andrea Hospital, Via di Grottarossa, 1035/1039, 00189 Rome, Italy; 6 Department of Neuroscience, Mental Health and Sense Organs, Sapienza University, Sant’Andrea Hospital, Via di Grottarossa, 1035/1039, 00189 Rome, Italy; 7 Flow Cytometry Unit, Clinical Laboratory, Sant’Andrea Hospital, Via di Grottarossa, 1035/1039, 00189 Rome, Italy; 8 UOSD Oncogenomica ed Epigenetica, IRCCS-Regina Elena National Cancer Institute, Via Elio Chianesi 53, 00144 Rome, Italy; 9 Scientific Direction, IRCCS Regina Elena National Cancer Institute, Rome, Italy; 10 UOSD SAFU, IRCCS-Regina Elena National Cancer Institute, Via Elio Chianesi 53, 00144 Rome, Italy

**Keywords:** Low T3 syndrome, Thyroid function, Coronavirus disease (COVID-19), Hematological malignancies, Differentially expressed genes, NanoString, CyTOF

## Abstract

**Background:**

Low T3 syndrome is frequent in patients admitted to intensive care units for critical illness and pneumonia. It has been reported also in patients with COVID-19, Hodgkin disease and chronic lymphocytic leukemia. We analyzed the clinical relevance of Low T3 syndrome in COVID-19 patients and, in particular, in those with associated hematological malignancies.

**Methods:**

Sixty-two consecutive patients, hospitalized during the first wave of SARS-CoV-2 outbreak in Sant’Andrea University Hospital in Rome, were subdivided in 38 patients (Group A), showing low levels of FT3, and in 24 patients (Group B), with normal FT3 serum values. During the acute phase of the disease, we measured serum, radiologic and clinical disease severity markers and scores, in search of possible correlations with FT3 serum values. In addition, in 6 COVID-19 patients, 4 with Low T3 syndrome, including 2 with a hematological malignancy, and 2 with normal FT3 values, we performed, high-dimensional single-cell analysis by mass cytometry, multiplex cytokine assay and gene expression profiling in peripheral blood mononuclear cells (PBMC).

**Results:**

Low FT3 serum values were correlated with increased Absolute Neutrophil Count, NLR and dNLR ratios and with reduced total count of CD3+, CD4+ and CD8+ T cells. Low FT3 values correlated also with increased levels of inflammation, tissue damage and coagulation serum markers as well as with SOFA, LIPI and TSS scores. The CyTOF analysis demonstrated reduction of the effector memory and terminal effector subtypes of the CD4+ T lymphocytes. Multiplex cytokine assay indicates that mainly IL-6, IP-10 and MCAF changes are associated with FT3 serum levels, particularly in patients with coexistent hematological malignancies. Gene expression analysis using Nanostring identified four genes differently expressed involved in host immune response, namely CD38, CD79B, IFIT3 and NLRP3.

**Conclusions:**

Our study demonstrates that low FT3 serum levels are associated with severe COVID-19. Our multi-omics approach suggests that T3 is involved in the immune response in COVID-19 and coexistent hematological malignancy and new possible T3 target genes in these patients have been identified.

**Supplementary Information:**

The online version contains supplementary material available at 10.1186/s12967-021-02805-6.

## Background

Zoonotic coronavirus has infected human populations three times in as many decades [1]. Coronavirus affect most the lung, but the disease can involve any organ during the viraemic phase. Previous observations indicated potential links between coronavirus infection and endocrine and metabolic alterations [2], mostly related to diabetes and hypertension. Few studies have analyzed other endocrinological alterations associated with coronavirus infections. The occurrence of chronic endocrine sequelae was evaluated in SARS survivors, showing evidences of hypopituitarism, including central hypocortisolism and central hypothyroidism [3]. Few autopsy studies are available regarding the occurrence of specific damages in endocrine tissues caused by coronaviruses [4, 5]. The Low T3 syndrome is also observed in COVID-19 patients, as reported in a recent study [6]. In addition, the Low T3 syndrome is considered a predictor of poor prognosis in chronic lymphocytic leukemia (CLL) [7], in Hodgkin disease (HD) [8] and in B cell Lymphoma [9].

The present study was performed to evaluate alterations in the thyroid function, associated with the acute phase of the SARS-CoV-2 virus infection and to correlate them with the severity of the disease. We also evaluated the clinical relevance of the Low T3 syndrome by gene signature analysis and immunological profiling in COVID-19 patients and in particular in those affected by coexistent hematological malignancies. We identified immunological alterations specifically associated with the occurrence of low FT3 serum values and a set of four genes that are deregulated in COVID-19 patients with Low T3 syndrome and with coexistent hematological malignancies.

## Methods

### Subjects recruitment

The research has been conducted at the Sant’Andrea University Hospital, designated as one of the referral Hospitals in Rome, Italy. During the first wave of SARS-CoV-2 outbreak, we examined a total of 62 hospitalized patients that were found to be positive for the presence of the SARS-CoV-2 virus in the nasal and oropharyngeal swabs, according to the WHO recommendations [10]. In particular, two real-time reverse-transcription polymerase chain reaction (rRT-PCR) detection kits have been used, namely the Allplex™ 2019-nCoV Assay (Seegene, Seul, Republic of Korea) and the RNA Detection kit (DAAN Gene Co. LTD, Guangzhou, Guandong, China), both CE-approved. The diagnosis has been considered positive in the presence of at least two of the three genes considered (E, RdRP and N genes) for the kit by Seegene and of the two genes considered (ORF1ab and N genes) for the kit by DAAN.

### Samples collection

In our COVID-19 patients we have evaluated the serum levels of FT3, FT4, TSH and of several severity markers during the acute phase of the disease, prior to any treatment. Blood samples have been drawn for blood cells count and measurement of the lymphocyte subpopulations by Flow Cytometric Analysis (FCA). The Procalcitonin (PCT) and the high-sensitive C Reactive Protein (hs-CRP) have been evaluated as markers of inflammation. The Prothrombin Time (PT), the Fibrinogen and the D-dimer have been measured as markers of coagulation. Tissue damages have been checked by measuring the Lactate DeHydrogenase (LDH), the Ferritin, the Creatinine, the total Bilirubin, the Creatine Kinase (CK), the Creatine Kinase-Myocardial Band (CK-MB) and the hypersensitive troponin I (hs-cTnI). Furthermore, we measured the immune marker interleukin-6 (IL-6).

A series of indexes have been calculated: including the neutrophil-to-lymphocyte ratio (NLR), as index of severe systemic inflammatory response in critically ill patients [11], the derived neutrophil-to-lymphocyte ratio (dNLR), previously used in cancer patients [12], the platelet-to-lymphocyte ratio (PLR), as an informative marker of acute inflammatory and prothrombotic states [13, 14] and the Lung Immune Prognostic Index (LIPI), as a prognostic index in metastatic non-small cell lung cancer [15, 16].

### Flow cytometric analysis (FCA)

FCA was performed in the automated AQUIOS CL® “load & go” flow cytometer (Beckman Coulter, Life Sciences Division, Indianapolis, USA), using florescent-labelled antibodies against the following cellular surface markers: CD45, CD19, CD3, CD4, CD8, CD14, CD15 and CD56. Briefly, 50 μl of whole blood was incubated with proper fluorescent-labelled antibodies for 30 min in dark at room temperature. 450 μl of 1× RBC FACS™ Lysing Solution (Becton, Dickinson and Company BD Biosciences, San Jose, USA) was added to the antibody-blood mixture and incubated for 15 min. at room temperature to remove red blood cells, which was ready for analysis on flow cytometer machine.

### The interleukin-6 (IL-6) assay

Interleukin-6 (IL-6) was measured using the IL-6 Human SimpleStep Elisa Kit (Thermo Fisher, Milan, Italy). All exams have been performed simultaneously to the measurements of thyroid function.

### Thyroid hormone function tests

The FT3, FT4 and TSH serum levels were assayed using a Chemiluminescent Microparticle Immunoassay (CMIA), using an immunoassay analyser (ARCHITECT i1000SR, Abbott Laboratories, Abbott Park, IL, USA) and specific diagnostic kits (ARCHITECT Free T3, FT4 and TSH assay, Abbott Laboratories, Abbott Park, IL, USA). Conventional reference intervals for FT3, FT4 and TSH were 1.71–3.71 pg/ml, 0.7–1.48 ng/dl and 0.35–4.0 µIU/ml, respectively.

### CT acquisition

Chest CT were obtained on a 128-slice scanner (GE Revolution EVO CT Scanner, GE Medical Systems, Milwaukee, WI, USA), with patients in supine position and during end-inspiration, without iodinated contrast medium injection. The following technical parameters were used: tube voltage: 120 kV; tube current modulation: 100–250 mAs; spiral pitch factor: 0.98; collimation width: 0.625. Images were reconstructed with a sharp convolution kernel (BONEPLUS) at a slice thickness of 1.25 mm.

### Quantification of lung CT lesions by TSS

DICOM data have been transferred onto a PACS workstation (Centricity Universal Viewer v.6.0, GE Medical Systems, Milwaukee, WI, USA) and independently evaluated by two expert radiologists, using a dedicated software (Thoracic VCAR v13.1, GE). Only imaging features related to COVID-19, according to the most recent literature [17, 18] have been considered valid for image analysis. For each patient, disease severity has been visually assessed through a “Total Severity Score” (TSS) [19]. In each lobe the percentage of parenchymal involvement was classified as none (0%), minimal (1–25%), mild (26–50%), moderate (51–75%) and severe (76–100%), with a score equal to 0, 1, 2, 3, 4, respectively. TSS, ranging from 0 to 20, was calculated by summing the scores of the five lobes.

### SOFA score

The Sequential Organ Failure Assessment (SOFA) score, a mortality prediction score based on the degree of dysfunction of six organ systems, has been calculated using the following variables: PaO_2_/FiO_2_ measured in mmHg, Platelets counted in ×10^3^/µl, the Glasgow Coma Scale from grade 0 to + 4, the bilirubin, measured in mg/dl, the mean arterial pressure in mmHg and the creatinine in mg/dl. The SOFA score has been recently used to evaluate the severity of the clinical conditions in COVID-19 patients [20].

### Subjects analyzed by mass cytometry, multiplex cytokine analysis and NanoString

In a small group of COVID-19 patients the impact of FT3 serum levels on the immunological and gene expression profiles has been analyzed using two very highly accurate and sensitive methods. In particular, peripheral blood mononuclear cells (PBMC) samples from COVID-19 patients with low and normal FT3 serum levels were immunophenotyped using mass cytometry and sera samples were characterized using multiplexed cytokine analysis. The mass cytometry, was performed using the Cytometry by Time-of-Flight (CyTOF) instrument, to evaluate and quantified the different subtypes of the circulating immune cells. In addition, a NanoString-based gene expression assay has been applied to get reliable, sensitive, and highly-multiplexed detection of mRNA targets. The blood samples have been obtained by 6 COVID-19 patients, four of which had Low T3 Syndrome and two had normal FT3 serum values. The epidemiological and clinical characteristic of these patients are reported in Table 1. Among the patients with COVID-19 and Low T3 Syndrome, a hematological malignancy was present in two cases and consisted in a Mixed Cellularity classical Hodgkin Lymphoma (MCcHL) (Sample # SAND-011) and in a chronic lymphocytic leukemia (Sample # SAND-006). Two samples from non-COVID-19 healthy donors (HC-02 and HC-06) have been used as controls.

**Table 1:** COVID-19 patients analyzed by CyTOF and Nanostring, divided according to their serum FT3 levels

Pts	Sex	Age	Neoplastic disease	Non-neoplastic diseases	FT3 (pg/ml)	FT4 (ng/ml)	TSH (µIU/ml)
A—Patients with Normal FT3 serum values							
Sand-100	M	68		Paroxysmal atrial fibrillation, peptic ulcers with anemia, COPD with pneumothorax in the past; monoclonal gammopathy	1.8	1.2	0.11
Sand-007	F	88		Hypertension	1.8	1.1	0.38
B—Patients with low FT3 serum values							
Sand-003	M	60		Hematoma of the right iliopsoas muscle (secondary to therapy with heparin); hemorrhagic liver cyst	1.3	0.8	0.77
Sand-006	M	74	Chronic lymphocytic leukemia (CLL)	Benign prostatic hypertrophy (BPH); pancreatic cyst	1.2	1.8	0.07
Sand-011	M	69	Mixed cellularity classical Hodgkin lymphoma (MCcHL)	Gallbladder stones; benign prostatic hypertrophy (BPH)	1	0.9	0.62
Sand-004	F	69		Encephalitis	< 1	1	0.2

### Isolation of peripheral blood mononuclear cells (PBMCs)

We have collected blood samples in vials supplemented with anticoagulants (EDTA). We have isolated PBMCs by density gradient centrifugation, using Lympholyte®-H Cell Separation Media (Cedarline Industries LTD, British Columbia, Canada). The blood samples have been diluted with 1:2 volumes phosphate-buffered saline (PBS), and gently layer the whole blood over top of the Lymphocyte (density gradient medium with ρ = 1.077 g/ml) making sure not mix the two layers, before centrifugation at 1600×*g* for 20 min at 4 °C in a swinging bucket rotor without brake. We carefully aspirated most of the upper layer, leaving the mononuclear cells at the interphase, transfer the mononuclear cells to a new 50 ml tube, fill the tube with PBS, and centrifuge at 1600×*g* for 10 min at 4 °C (twice). After removal of most of the platelets, we have stored the PBMC in two different storage media. Prior to storage, the batches have been aliquoted in numbers and volumes appropriate for each protocol. A suspension of 10^7^ PBMCs/ml has been preserved in freezer medium, and a final concentration of 2 × 10^6^ PBMCs/ml was resuspended in QIAzol Lysis Reagent (QIAGEN, Manchester, UK).

### Immune phenotyping by CyTOF and data analysis

Antibody staining Maxpar Direct Immune Profiling Assay was used to characterize immune phenotyping of PBMC samples, according to the method previously reported [21]. Briefly, cryopreserved PBMC were thawed, treated with DNAsiI (Roche), counted and cell viability was determined. Cells were washed in Cell Staining Buffer (CSB) and 3 × 106 cells were resuspended in 50 μl of the same buffer. Samples were treated with Human TruStain FcX for blocking FC receptors. Then samples were diluted and added to the dry antibody tubes for antibody staining. After a 30-min incubation, the cells were washed twice in CSB and fixed in 1.6% paraformaldehyde. Then, cells were resuspended in 1 ml of the 125 nM Cell-ID Intercalator-Ir and freezed at − 80 °C. Before data acquisition, fixed cells were washed twice in CSB, twice in Maxpar Cell Acquisition Solution (CAS) and counted. Cell were resuspended in CAS containing 0.1× EQ™ Four Element Calibration Beads with a final concentration of 1 × 106 cells/ml. Samples were filtered through a 35 μm cell strainer. Sample acquisition was performed using Helios system. Following antibodies were analyzed: CD45, CD196/CCR6, CD123, CD19, CD4, CD8a, CD11c, CD16, CD45RO, CD45RA, CD161, CD194/CCR4, CD25, CD27, CD57, CD183/CXCR3, CD185/CXCR5, CD28, CD38, CD56/NCAM, TCRgd (B1), CD294, CD197/CCR7, CD14, CD3, CD20, CD66b, HLA-DR, IgD, CD127, live/dead intercalator-103Rh.

Data were analyzed by Maxpar® Pathsetter™ automated package (Fluidigm, South San Francisco, CA, USA), powered by GemStone™ 2.0.41 (Verity Software House, Topsham, ME, USA). Data were normalized using the CyTOF Software v.6.7.1016, with a Cleanup PSM model leveraging Gaussian pulse-processing parameters to eliminate unwanted events. Normalized files contain only intact live singlet cells. Automated analysis identifies and labels the major immune cell populations in sample files. This system is integrated with dimensionality-reduction mapping known as Cauchy Enhanced Nearest-neighbor Stochastic Embedding (Cen-se′™), which generates a visual display of high-dimensional data labeled with the major cell populations. The high-dimensional Cen-se′™ (next-gen t-SNE) map has been used to identify and visualize all populations of immune cells in our samples [22].

### Multiplex cytokine immunoassay

The Bio-Plex Pro Human Cytokine 27-Plex Immunoassay (Bio-Rad Laboratories S.r.l. Segrate, Milan, Italy) has been used to characterized Cytokine Immunoassay of human sera samples. Samples were prepared following the manufacturer’s instructions and quantified in according to the generation of the standards curves over a broad range for each target. The magnetic bead-based multiplex assay system allows detection and quantification of multiple cytokines from a single sample of plasma. Primary antibodies are coupled to the magnetic beads, detection proceeds with secondary antibody and streptavidin-PE. Following Cytokine were analyzed: FGF basic, Eotaxin, G-CSF, GM-CSF, IFN-γ, IL-1β, IL-1ra, IL-2, IL-4, IL-5, IL-6, IL-7, IL-8, IL-9, IL-10, IL-12 (p70), IL-13, IL-15, IL-17, IP-10, MCP-1 (MCAF), MIP-1α, MIP-1β, PDGF-BB, RANTES, TNF-α, VEGF. Sample acquisition has been performed using the Bio-plex Manager MP software with the MagPix instrument. Data were analized with xPONENT software.

### NanoString analysis

Transcriptome analysis is based on the nCounter® PanCancer human IO 360™ Panel and NanoString platform (NanoString, Seattle, WA, USA). This panel allows simultaneous analysis of 770 genes involved in the immune response in cancer. We compared the NanoString data from our six COVID-19 patients with two healthy control donors. Principal component analysis (PCA) and ANOVA analysis are performed using Partek Genomic Suite (Partek Incorporated, St. Louis, MO, USA). PCA was used to characterize samples based on their gene expression profiles. ANOVA analysis is used to identify differentially expressed genes (significant p-value < 0.05) and samples are clustered by hierarchical clustering.

### Statistical analysis

Descriptive statistical analysis is performed on raw data after a one-sample Kolmogorov–Smirnov test [23]. Due to low number of patients an 1% significance level is accepted to reject the null hypothesis. The results are expressed as means ± SD. A two-tailed *P* value of 0.05 or less has been used as a criterion to indicate statistical significance. NS = not significant. Multivariate linear regression analysis is performed using FT3 serum value as a dependent variable. Data have been statistically analyzed using the GraphPad Prism software (version 8.4.1) (GraphPad Software, San Diego, CA, USA).

### Ethical approval

A written informed consent was obtained by participant to the study. The study was approved by our Institutional Ethical Committee (University La Sapienza of Rome, Italy) (RIF. CE 5773_2020, Prot. # 52SA_2020, and its subsequent substantial amendment RIF. CE 5773_2020, Prot. # 171SA_2020), on the basis that it complied with the declaration of Helsinki and that the protocol followed existing good clinical practice guidelines.

## Results

### Epidemiological, anamnestic and clinical data

A total of 62 consecutive patients, referred to our hospital for COVID-19 and identified as laboratory-confirmed SARS-CoV-2 infection, have been included in the study. They are 29 men and 33 women. Their median age is 68.4 years (ranging from 29 to 100 years) (Table 2). Most of them had underlying diseases (54 [87%]), including diabetes (32 [51%]), hypertension (40 [64.5%]), cardiovascular disease (27 [43%]), COPD (12 [34%]) and malignancy (9 [14%]).

**Table 2:** Epidemiological and anamnestic data of our COVID-19 patients

Characteristics	All patients (n = 62)	Group A (n = 38)	Group B (n = 24)	*P* value
Age, years	68.4 (29–100)	70.5 (32–100)	65.5 (29–87)	NS
Sex				
Men	29 (47%)	19 (50%)	10 (42%)	NS
Women	33 (53%)	19 (50%)	14 (58%)	NS
Current smoking	22 (35.5%)	10 (26.3%)	12 (50%)	0.03
Comorbidity (at least one)	54 (87.1%)	33 (86.8%)	21 (87.5%)	NS
Diabetes	32 (51.6%)	24 (63.2%)	8 (33.3%)	0.03
Hypertension	40 (64.5%)	18 (47.4%)	12 (50.0%)	NS
Cardiovascular disease	27 (43.5%)	19 (50.0%)	8 (33.3%)	NS
Chronic obstructive pulmonary disease	21 (33.9%)	11 (28.9%)	10 (41.7%)	NS
Malignancy	9 (14.5%)	6 (15.8%)	3 (12.5%)	NS
Multiple sclerosis	1 (1.6%)	0 (0%)	1 (4.2%)	NS

Data are reported for all patients and for patients with low FT3 values (Group A), compared to those with normal FT3 values (Group B). The *p* values are indicated as a measure of the levels of significance of the differences among the two groups

The epidemiological, anamnestic and clinical data regarding the patients are listed in Table 2. Patients have been divided into two groups according to their FT3 values. Thirty-eight patients are classified in Group A because of a low FT3 serum value (≤ 1.7 pg/ml, or ≤ 3.2 pmol/l). Twenty-four patients are included in the Group B because they show a normal FT3 serum value (between 1.7 pg/ml and 3.71 ng/ml or between 3.2 and 12.8 pmol/l). In Group A there are equal numbers of males and females (M/F ratio = 1.0), while in Group B the proportion of women is slightly higher (M/F ratio = 0.7). A malignant lesion was reported in both group at a similar rate, namely 15.8% in Group A compared to 12.5% in Group B. The mean values of all the severity markers of the disease that we have measured are reported in Table 3.

**Table 3:** Severity indexes of the disease in our COVID-19 patients

Severity indexes	COVID-19 patients			
	All patients (n = 62)	Group A (n = 38) FT3 ≤ 1.7 pg/ml	Group B (n = 24) FT3 > 1.7 pg/ml	*P* value
Thyroid function				
FT3 (pg/ml)	1.7 ± 0.6	1.2 ± 0.2	2.1 ± 0.6	≪ 0.005
FT4 (ng/dl)	1.1 ± 0.2	1.0 ± 0.3	1.1 ± 0.1	NS
TSH (µIU/ml)	1.0 ± 0.9	0.7 ± 0.8	1.3 ± 1.1	0.01
Hemocromocytometric parameters				
RBC (×10^6^cells/µl)	4.0 ± 0.7	3.9 ± 0.7	4.2 ± 0.7	0.06
HGB (g/dl)	11.9 ± 1.9	11.5 ± 1.9	12.5 ± 1.9	0.03
WBC (×10^3^cells/µl)	8.6 ± 3.9	9.2 ± 4.4	7.7 ± 2.9	0.07
ANC (×10^3^cells/mm^3^)	6.5 ± 3.4	7.6 ± 3.5	5.0 ± 2.5	0.002
ALC (×10^3^cells/mm^3^)	1.4 ± 1.6	1.1 ± 1.9	1.7 ± 0.8	0.09
NLR	8.2 ± 7.9	11.2 ± 8.7	3.4 ± 2.8	0.00004
dNLR	5.8 ± 4.4	7.4 ± 4.8	3.3 ± 1.6	0.0001
Lymphocytes subpopulations				
CD45+ (cells/µl)	1034 ± 1147.6	902.4 ± 1235.3	1341.3 ± 871.6	NS
CD19+ (cells/µl)	251.9 ± 973.4	293.4 ± 1164.2	155.9 ± 135.0	NS
CD3+ (cells/µl)	629.9 ± 500.8	467.1 ± 234.9	1006.4 ± 721.5	0.0001
CD45+CD3+CD8+ (cells/µl)	211.8 ± 169.4	174.9 ± 134.5	297.2 ± 212.1	0.007
CD45+CD3+CD4+ (cells/µl)	402.2 ± 366.2	282.2 ± 158.7	686.2 ± 513.9	0.0005
CD4+/CD8+ ratio	2.4 ± 1.6	2.22 ± 1.61	2.88 ± 1.6	0.09
CD3−CD16+CD56+ (cells/µl)	115.3 ± 83.5	99.7 ± 60.7	150.9 ± 115.7	0.02
Serum markers of inflammation				
APC (×10^3^cells/µl)	215.1 ± 111.2	186.0 ± 96.4	261.1 ± 119.1	0.005
PLR	231.4 ± 136.4	254.0 ± 131.2	195.5 ± 139.3	0.05
PCT (ng/ml)	3.3 ± 17.2	4.8 ± 20.9	0.2 ± 0.3	NS
hs-CRP (mg/dl)	10.0 ± 13.1	12.7 ± 14.5	5.1 ± 8.0	0.006
Serum markers of tissue damages				
CK (U/l)	81.0 ± 98.7	82.1 ± 104.1	79.4 ± 91.6	NS
CK-MB (ng/ml)	1.2 ± 1.4	1.4 ± 1.7	1.1 ± 0.8	NS
Bilirubin (mg/dl)	0.6 ± 0.6	0.7 ± 0.7	0.5 ± 0.2	NS
hs-cTnI (pg/ml)	41.0 ± 93.3	59.8 ± 110.8	9.7 ± 16.5	0.02
Creatinine (mg/dl)	1.2 ± 1.4	1.4 ± 1.7	0.9 ± 0.4	0.09
Ferritin (ng/ml)	769.2 ± 743.2	1085.6 ± 950.8	495.5 ± 482.8	0.009
LDH (U/l)	300.1 ± 101.8	328.2 ± 105.7	253.6 ± 79.1	0.002
Serum coagulation markers				
Fibrinogen (mg/dl)	555.2 ± 251.8	602.4 ± 269.0	476.5 ± 202.9	0.03
PT (s)	13.8 ± 4.7	13.3 ± 1.7	14.6 ± 7.2	NS
D-dimer (ng/ml)	857.8 ± 1055.8	1038.6 ± 964.6	582.3 ± 1050.1	0.04
Serum immunological markers				
IL-6 (pg/ml)	181.3 ± 456.7	232.1 ± 580.6	103.9 ± 94.4	NS
Severity scores of lung damage				
LIPI score	1.5 ± 0.6	1.8 ± 0.4	1.2 ± 0.6	0.00003
Radiological severity scores of lung damage				
TSS score	8.1 ± 4.3	9.6 ± 3.9	5.7 ± 3.9	0.0004
Clinical severity score				
SOFA score	2.8 ± 1.8	3.3 ± 1.7	2.3 ± 1.7	0.001

Data are reported for all patients and for patients with low FT3 values (Group A), compared to those with normal FT3 values (Group B). For each parameter the mean values ± the standard deviations are reported. The *p* values are indicated as a measure of the levels of significance of the differences among the two groups

*NS* not significant

### Thyroid function tests

In patients of Group A, the reduced levels of the serum FT3 values is observed in association with the reduction in the mean TSH serum levels. In 19 cases of Group A, the TSH serum levels are suppressed under the lower normal limit, and in two of them there is also a reduction in the FT4 serum levels. There is a clear correlation between reduced levels of FT3 and reduced levels of TSH (Additional file 1: Fig. S1).

### Hemocromocytometric parameters

**Fig. 1:**
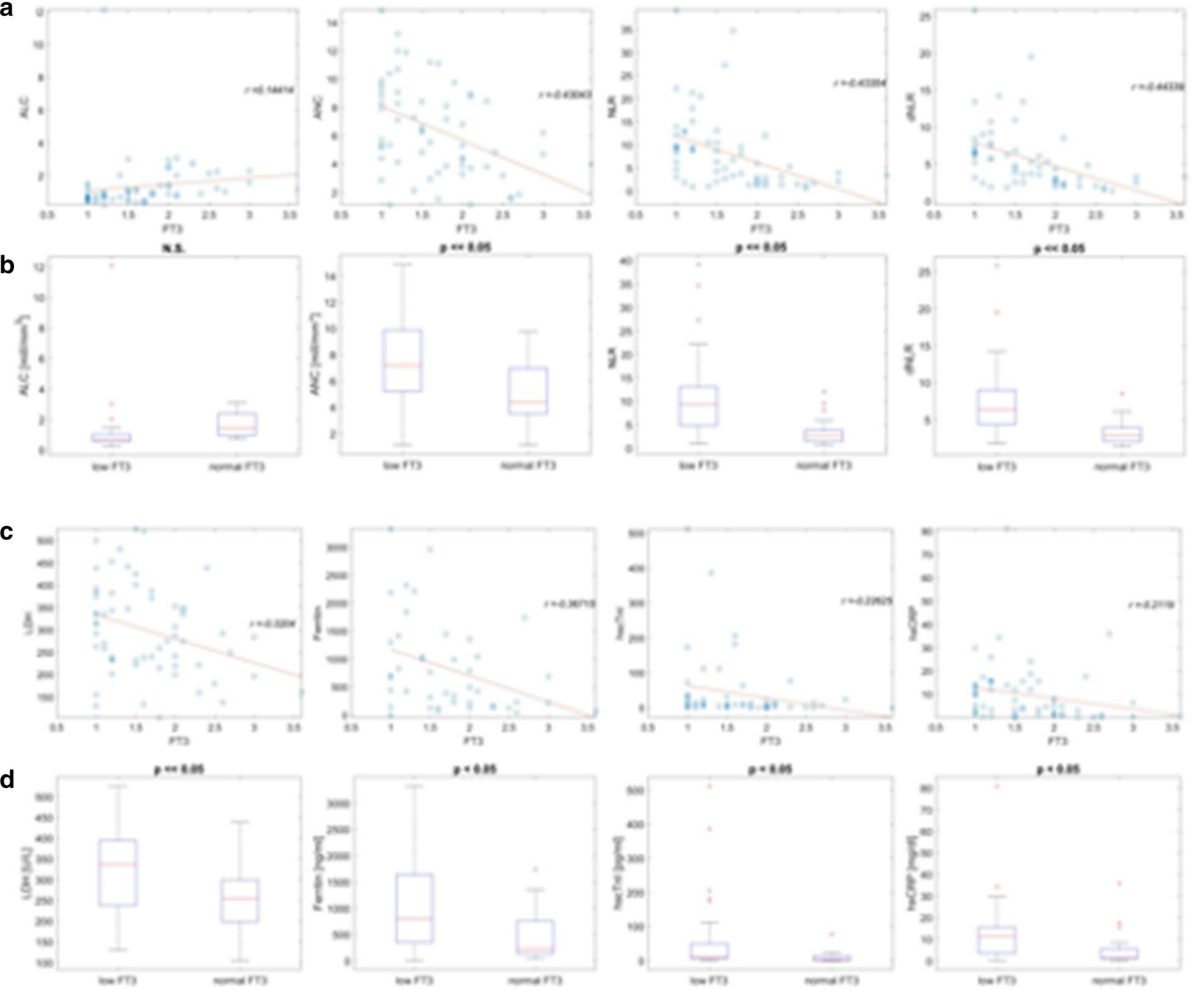
Correlations of FT3 serum values in COVID-19 patients with blood cells and disease markers. **a** Graphic correlations, based on cartesian coordinate system, of ALC, ANC, NLR and dNLR with the serum FT3 values. **b** Box plots of ALC, ANC, NLR and dNLR in COVID-19 patients with low serum FT3 values (Group A), compared to normal FT3 values (Group B). **c** Graphic correlation between LDH, Ferritin, hs-cTnI and hs-CRP and serum FT3 values. **d** Box plots of LDH, Ferritin, hs-cTnI and hs-CRP in COVID-19 patients with low serum FT3 values (Group A), compared to normal FT3 values (Group B). The corresponding correlation coefficient *r* for each scatter plot and the *p* value for each box plot are indicated. *ALC* absolute lymphocyte count, *ANC* absolute lymphocyte count, *NLR* neutrophil-to-lymphocyte ratio, *dNLR* derived NLR. The corresponding correlation coefficient *r* for each scatter plot and the *p* value for each box plot are indicated

The mean blood counts of our COVID-19 patients, measured on admission, show normal values of red blood cells (RBC), white blood cell (WBC) and platelets (PLT) (Table 3). The mean values of both NLR and dNLR are increased. When we comparatively examined the results in the two groups, we find that a statistically significant difference (*p* ≤ 0.05) is present in many parameters of the CBC (Table 3; Fig. 1). The WBC and the Absolute Neutrophils Count (ANC) are increased in Group A compared to Group B. Conversely, the absolute count of RBC and the value of hemoglobin are reduced in Group A compared to Group B. The Absolute Lymphocyte Count (ALC) is reduced too in patients of Group A compared to those in Group B, although with no statistically significance. As a consequence, we observed a higher value of both NLR and dNLR in Group A compared to Group B. Hemocromocytometric analysis indicates a clear correlation between the FT3 serum levels and the NLR and dNLR (Fig. 1a, b). Therefore, the low FT3 values appear to be correlated with mild anemia and, in a strong and highly significant manner, with increased ANC, NLR and dNLR.

### Serum markers of inflammation

We have analyzed the Absolute Platelet Count (APC), the PLR, the PCT and the hs-CRP as markers of systemic inflammation. The mean value of all of them are reported in Table 3. Patients in Group A show a slightly reduced APC values, compared to those in Group B, with an increased PLR (Additional file 2: Fig. S2). In both cases the difference is statistically significant. Low FT3 serum levels are associated with a significant reduction of both PLT and PLR.

The PCT is slightly elevated in all COVID-19 patients examined. A minor and not significant difference is observed in the two Groups. The hs-CRP is highly elevated in all patients and it is much higher in Group A compared to Group B. The hs-CRP serum levels appear to be inversely correlated to the FT3 serum levels (Table 3; Fig. 1c, d).

### Serum markers of tissue damages

The serum levels of several markers of tissue damages have been assessed in our two Groups of patients (Table 3). No significant differences are visible between the two Groups in serum levels of CK, CK-MB, total Bilirubin, aspartate aminotransferase (AST) and alanine aminotransferase (ALT). Their medium values are all in the normal ranges. The serum levels of hs-cTnI and of LDH appear to be inversely correlated with the serum FT3 levels in our COVID-19 patients (Fig. 1c, d). The serum levels of hs-cTnI are markedly elevated in 10 out of 38 patients (26.3%) in Group A, in whom the diagnosis of virus-related cardiac injury has been made, and in only 1 patient out of 24 (4.2%) in Group B. The mean serum levels of hs-cTnI are higher in Group A, compared to Group B. Creatinine, is only slightly higher in Group A, compared to Group B. A marked difference between the two Groups is also visible in the serum levels of Ferritin and LDH. Mean value of serum Ferritin, in fact, is rather high in all COVID-19 patients and it is much higher in Group A, compared to Group B. Another marker of tissue damage that is substantially increased in all COVID-19 patients is the serum LDH. LDH serum values are higher in Group A, compared to values in Group B. Our results indicate that in COVID-19 patients low FT3 values are associated with increased serum levels of tissue damage markers, especially the LDH, Ferritin and hs-cTnI (Table 3; Fig. 1c, d) (Additional file 3: Fig. S3a).

### Serum coagulation markers

Then we checked whether the FT3 values were associated with alterations in the serum levels of coagulation markers, namely the PT, the Fibrinogen and the D-Dimer. PT is above the normal range (PT > 13 s.) in 18 out of 32 patients (56.3%) in Group A and in 10 out of 24 patients (41.7%) in Group B. Mean Fibrinogen value is high in all our COVID-19 patients. Its levels are different in the two Groups, with higher values in Group A patients compared to Group B. The mean serum levels of the D-dimer are also increased in our COVID-19 patients and much higher values are detected in Group A patients, compared to Group B. We detected a correlation between the FT3 values and the Fibrinogen (Additional file 2: Fig. S2) and the Fibrinogen values are higher in patients with low FT3 serum values (Table 3).

### Serum IL-6 values

We found values of IL-6 above the upper normal limit in almost all patients, with large variability among them. No correlation is detectable between FT3 values and IL-6. The mean IL-6 values in Group A is slightly elevated compared to patients in Group B, but the difference is not significant.

### Flow cytometric analysis (FCA)

**Fig. 2:**
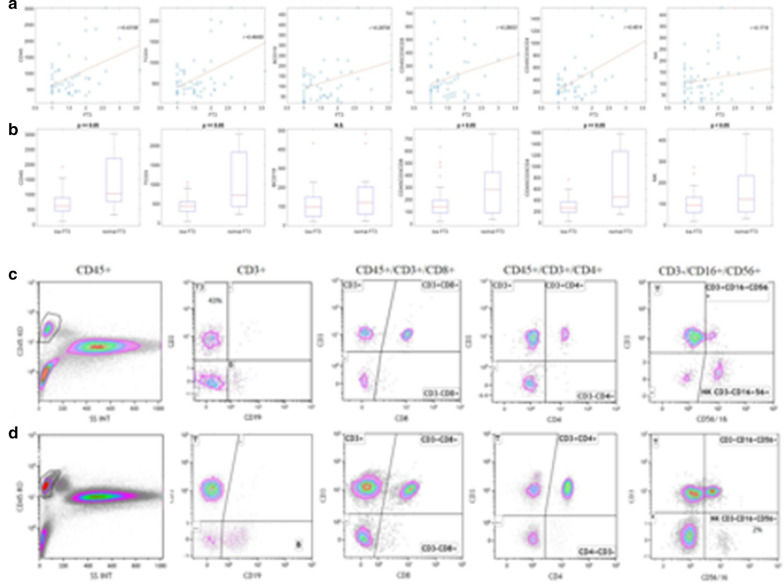
Correlations of FT3 serum values in COVID-19 patients with lymphocyte subpopulations. **a** Graphic correlation between CD45+, CD3+, CD19+, CD8+, CD4+ and NK cells and serum FT3 values. **b** Box plots of CD45+, CD3+, CD19+, CD8+, CD4+ and NK cells in COVID-19 patients with low serum FT3 values (Group A), compared to normal FT3 values (Group B). The corresponding correlation coefficient *r* for each scatter plot and the *p* value for each box plot are indicated. **c** Representative images of Flow Cytometric Analysis (FCA) in COVID-19 patients using fluorescently-tagged monoclonal antibodies specific for CD45+, CD19+, CD8+, CD4+ and CD16+/CD56+ in COVID-19 in patients with low serum FT3 values (Group A). **d** FCA using fluorescently-tagged monoclonal antibodies specific for CD45+, CD19+, CD8+, CD4+ and CD16+/CD56+ in COVID-19 in patients with normal serum FT3 values (Group B)

One of the hallmarks of the COVID-19 is represented by the marked lymphocytopenia [24]. To further investigate the relationship between serum FT3 values and lymphocytes, we performed FCA to analyze the composition of the lymphocyte subpopulations in the peripheral blood of our patients and the results are shown in Table 3. We observed a marked reduction affecting many lymphocyte subpopulations in both groups of patients. The absolute total count of T lymphocytes and both CD8+ and CD4+ are markedly reduced and such reduction is more severe in the CD4+ subpopulation. The NK cells are only slightly reduced. We didn’t observe any significant variation in the B lymphocytes. We detected a direct correlation between serum FT3 levels and CD3+, CD45+CD3+CD8+ and with CD45+CD3+CD4+ lymphocytes (Fig. 2a, b). The analysis of the FCA results in the two groups of patients shows noticeable differences (Table 3; Fig. 2). Patients in Group A are characterized by a more severe reduction in the absolute total count of T lymphocytes, compared to patients in Group B. Patients with low FT3 levels show also a severe reduction in both CD4+ and in CD8+ lymphocytes. However, the reduction affects more the CD4+ subpopulation, with a reduced CD4+/CD8+ ratio observed in Group A, compared to Group B. In addition, a more significant reduction is observed in NK subpopulation in patients belonging to Group A compared to those belonging to Group B. No differences between the two groups are visible in the absolute count of B lymphocytes. These results indicate a strong correlation between the reduction of FT3 serum levels and the reduction of serum T lymphocytes, especially affecting the CD4+ subpopulation (Fig. 2). FCA analysis of the different lymphocyte subpopulations, in representative cases of the two Groups of patients are shown (Fig. 2c, d) (Additional file 3: Fig. S3b).

### Lung Immune Prognostic Index

Since both dNLR and LDH are inversely correlated with FT3 values in our COVID-19 patients, we checked whether the combination of both of them in the LIPI score could be also correlated with low FT3 values. LIPI score range from 0 to 2. The highest LIPI score of 2 is detected in 29 patients out of 38 (76.3%) in Group A, compared to 7 patients out of 24 (20.8%), in Group B. A higher LIPI score is observed in Group A, compared to Group B and, therefore, it appears to be strongly correlated with low FT3 values (Table 3).

### Clinical severity scores

The mean SOFA score measured in our COVID-19 patients is reported in Table 3. Patients in Group A show a higher SOFA score compared to those in Group B. Low FT3 values are clearly associated with higher SOFA score.

### Radiological severity scores of lung damage

**Fig. 3:**
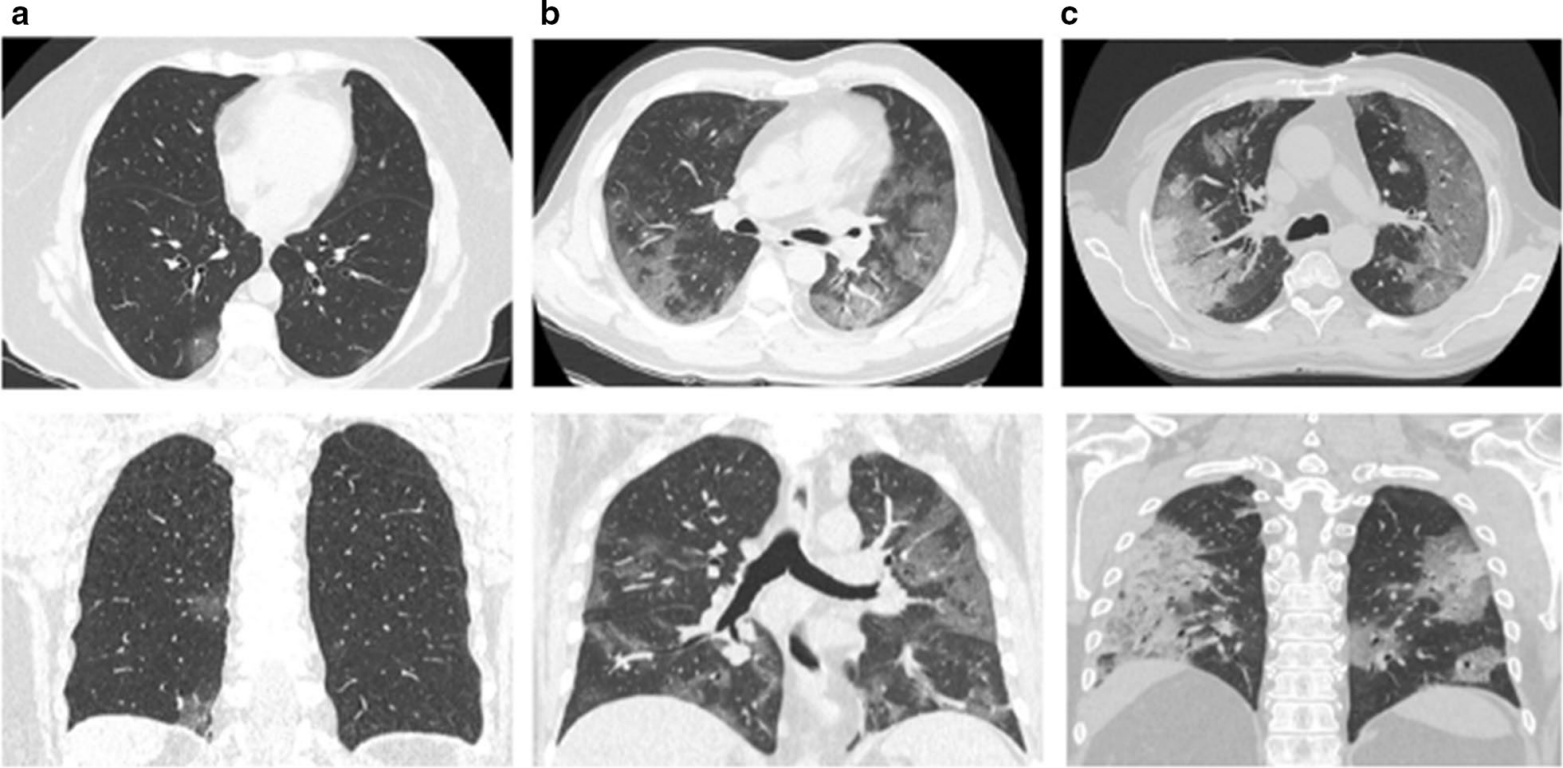
COVID-19 interstitial lung pneumonia radiologic images in patients with or without Low T3 syndrome. Representative CT images of different degrees of severity in a patient with normal FT3 value (**a**) and in two patients with low FT3 levels (**b**, **c**). **a** Minimal involvement of right inferior lobe. Two ground glass opacity in the subpleural space. **b** Moderate involvement of both lungs. Diffuse ground glass opacities, with peripheral distribution and subsegmental vessel enlargement. **c** Severe involvement of both lungs. Diffuse ground glass opacities, especially with peripheral distribution. Consolidations are also detected, mostly with peripheral distribution, in right upper lobe posterior segments. For each case both axial and coronal sections are shown

The median TSS, evaluated on admission, are higher in patients with low FT3 values compared to those observed in patients with normal FT3 values. Therefore, on CT visual quantitative analysis, the most severe degrees of acute lung inflammation appear to be strongly associates with low FT3 values. Representative CT images of patients with mild, moderate and severe degrees of lung inflammation are reported (Fig. 3).

### Multivariate analysis

**Fig. 4:**
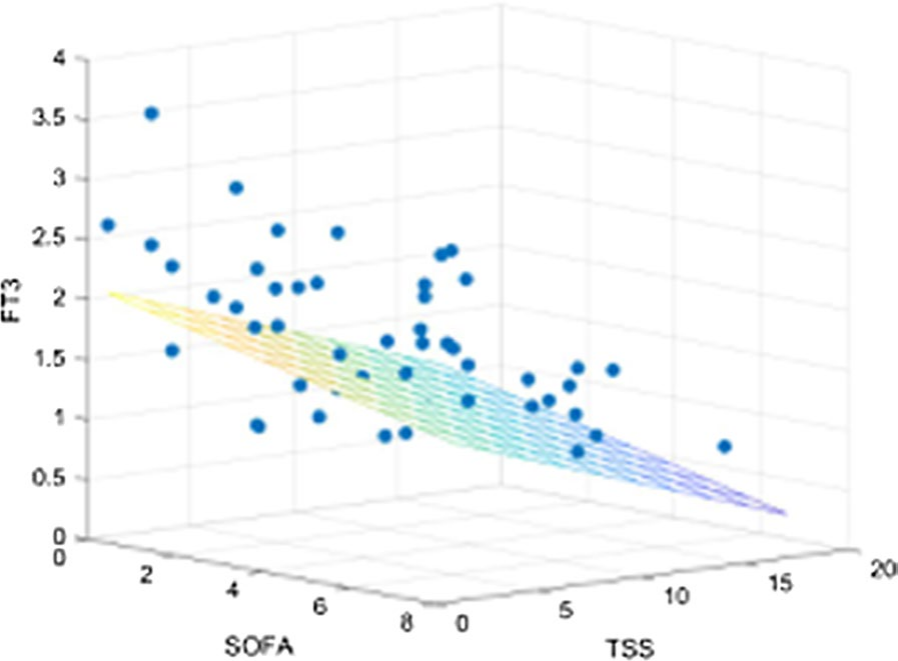
Curve-fit in 3D Scatter plot diagram showing the correlation among FT3 serum levels, the clinical SOFA score and the radiological TSS in COVID-19 patients

Variables with a *p* value of < 0.05 by univariate analysis were entered into the multivariate regression model. The multivariate linear regression analysis shows that there is a very high degree of correlation between FT3 values and hemocromocytometric and FCA parameters, including WBC, ANC, ALC and the T cells expressing the CD3+, CD4+CD8+, with a very high values of the coefficient of determination *R*^2^ and of the significance (Table 4). When we graphically correlated the serum FT3 values with the clinical and radiological scores, namely the SOFA and the TSS, in a 3D scatter plot diagram, we observed a clear correlation among them (Fig. 4).

**Table 4:** Multivariate analysis of the severity disease indexes that showed the highest significant differences at the univariate analysis

Severity disease indexes	*R*^2^	95% confidence interval	Standard error	*P* value
Hemocromocytometric and flow cytometry				
WBC	0.99	− 0.26 to 0.66	0.23	< 0.0001
ANC	0.99	− 0.73 to 0.21	0.23	
ALC	0.97	− 0.73 to 0.25	0.24	
CD3+	0.99	− 0.01 to 0.01	0.01	
CD45+CD3+CD8+	0.96	− 0.01 to 0.01	0.01	
CD45+CD3+CD4+	0.99	− 0.01 to 0.01	0.01	
Markers of inflammation/coagulation				
APC	0.37	0.01 to 0.01	0.01	< 0.0001
PLR	0.42	− 0.01 to − 0.01	0.01	
hs-CRP (mg/dl)	0.10	− 0.01 to 0.01	0.01	
Fibrinogen (mg/dl)	0.23	− 0.01 to 0.01	0.01	
D-dimer (ng/ml)	0.09	− 0.01 to 6.6	6.98	
Markers of tissue damages				
hs-cTnI (pg/ml)	0.14	− 0.01 to 0.01	0.01	0.0001
Creatinine (mg/dl)	0.15	− 0.38 to 0.40	0.19	
Ferritin (ng/ml)	0.44	− 0.01 to 0.01	0.01	
LDH (U/l)	0.47	− 0.01 to 0.01	0.01	

The coefficient of determination *R*^2^, the 95% confidence interval, the standard error and the *p* values are reported

### CyTOF analysis

**Fig. 5:**
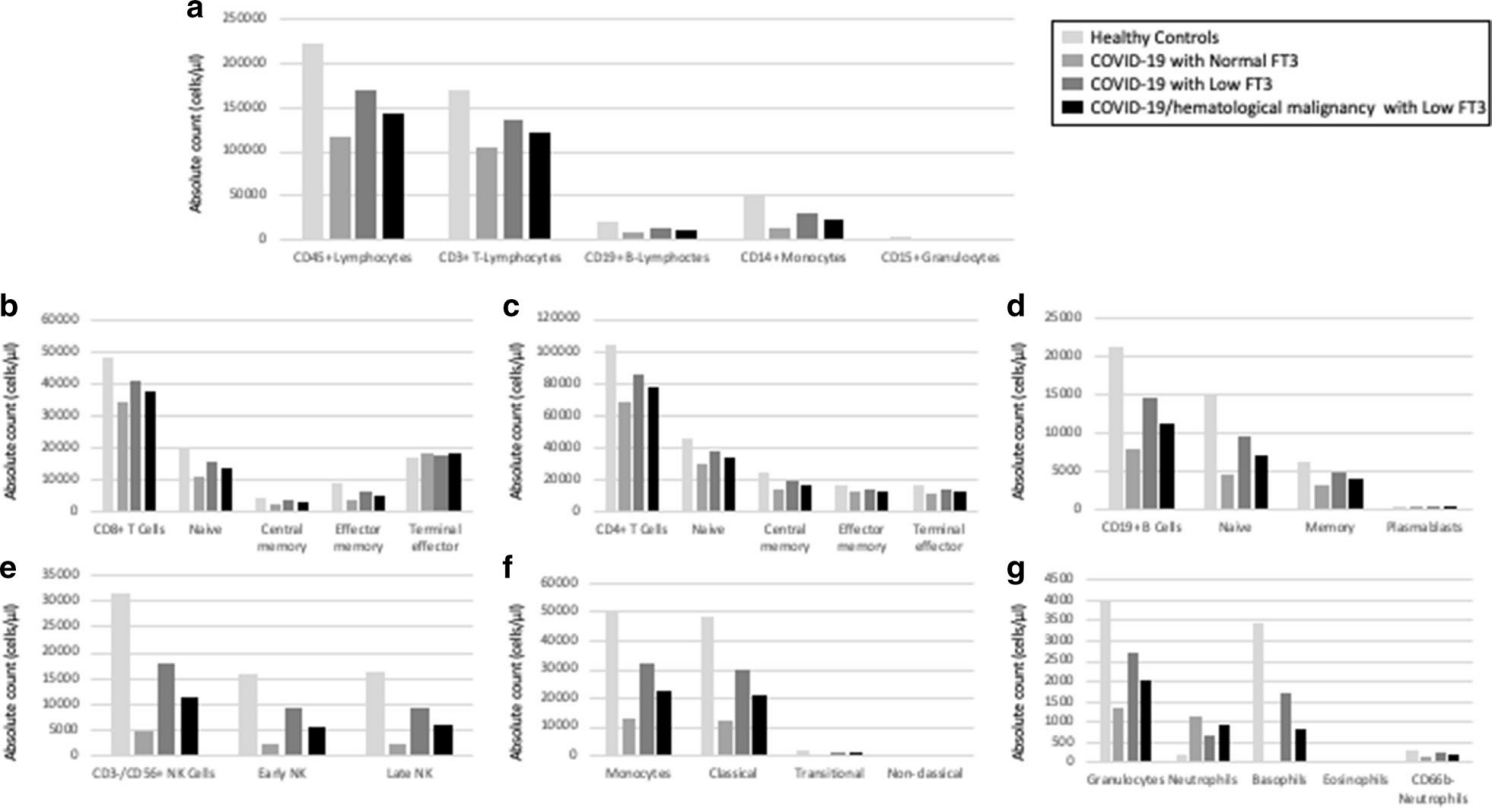
Bar charts of the results of CyTOF analysis, performed on our COVID-19 patients. Results are expressed as mean values of the percentage of live cells and refers to healthy controls (light gray bars), COVID-19 patients with normal FT3 serum values (gray bars), COVID-19 patients with low FT3 serum values (dark gray bars) and COVID-19 patients with hematological malignancy and low FT3 serum values (black bars). **a** Absolute count of lymphocytes (CD45+), T-lymphocytes (CD3+), B-lymphocytes (CD19+), monocytes (CD14+), of Dendritic cells and of Granulocytes (CD15+); **b** absolute count of T-lymphocytes (CD3+/CD8+) and of their subtypes; **c** absolute count of T-lymphocytes (CD3+/CD4+) and of their subtypes; **d** absolute count of B-lymphocytes (CD19+) and their subtypes; **e** Absolute count of NK-cells (CD3-/CD56+) and their subtypes; **f** absolute count of monocytes (CD14+) and their subtypes; **g** absolute count of Granulocytes (CD15+) and their subtypes

**Fig. 6:**
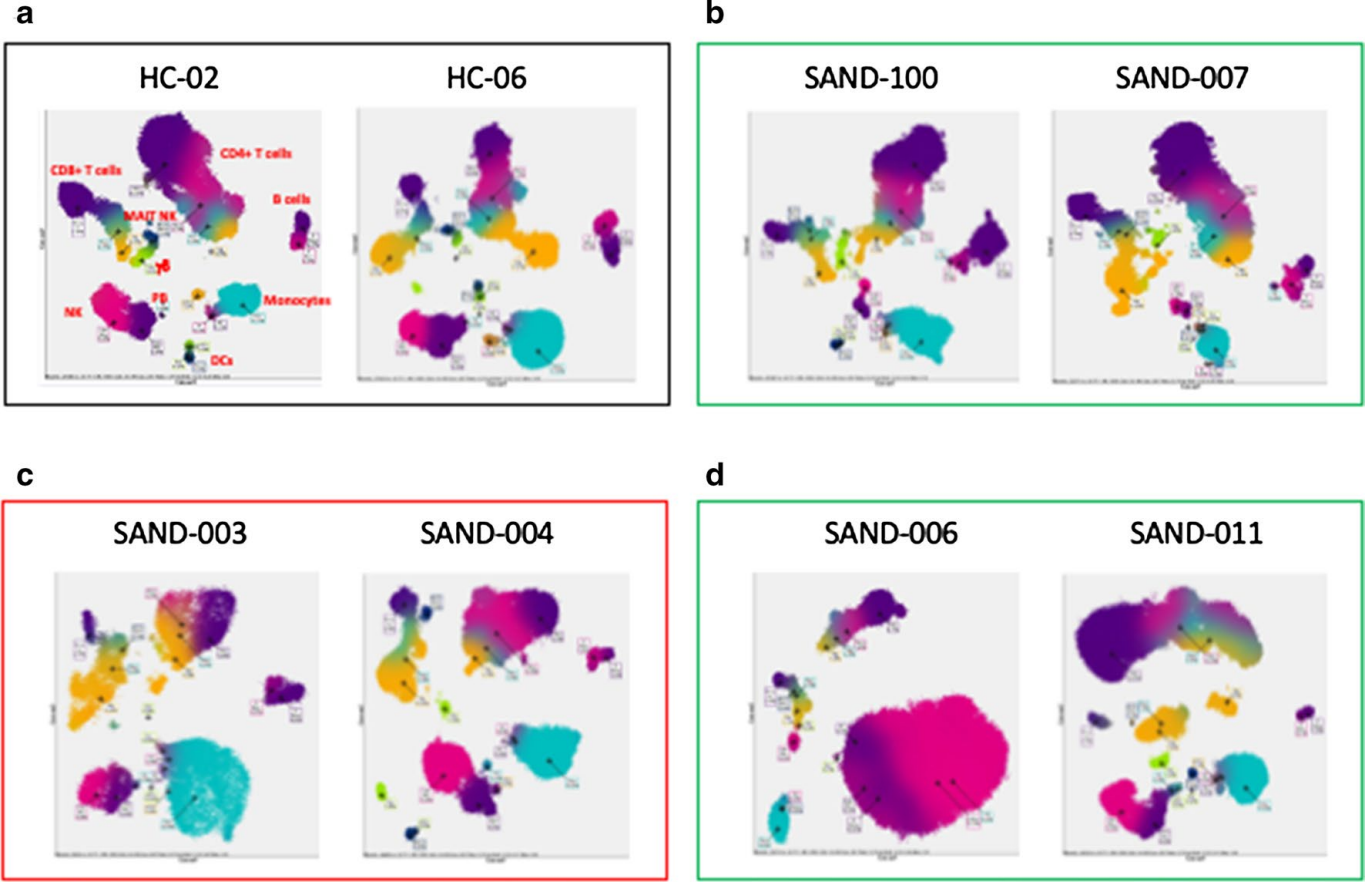
Pathsetter Cen-se′™ plot of the CyTOF data of healthy controls (HC), COVID-19 patients/normal FT3, COVID-19 patients/low FT3 and COVID-19 with hematological malignancy/low FT3 serum values. COVID-19 patients exhibit peculiar differences in the quantity of the different immune cell subpopulations in relation to FT3 serum levels and to the presence of hematological malignancy. **a** Healthy controls (HC); **b** COVID-19 patients with normal FT3 serum values; **c** COVID-19 patients with low FT3 serum values; **d** COVID-19 with hematological malignancy and with low FT3 serum values

There is an overall concordance among the results obtained by CyTOF analysis, and those obtained by FCA, with some minor differences (Fig. 5). In agreement with the results of FCA, we observed a reduction in the total lymphocytes (CD45+) in patients with Low T3 syndrome, compared to patients with normal T3 serum levels. Such reduction is not observed in COVID-19 patients with low FT3 serum values who are affected by coexistent hematological malignancy. The B lymphocytes (CD19+) are not increased in all COVID-19 patients with low FT3 serum values but only in those that have coexistent hematological malignancy (Fig. 5d). CyTOF analysis indicates that, in disagreement with FCA results, low FT3 serum values are associated with an increase in the NK cells (CD3−/CD56+), both the early and late subtypes (Fig. 5e), and an increase in the Monocytes (CD14+) too, that especially affects the classical subpopulation (Fig. 5f). These alterations are not seen in COVID-19 patients with hematological malignancies. Compared to COVID-19 patients with normal FT3 serum values, patients with low FT3 serum values show reduced CD4+ and CD8+ T lymphocytes at CyTOF analysis and these results are in agreement with those observed at FCA. We don’t observe any significant difference at CyTOF analysis in the absolute count of Treg, γδ T cells and of dendritic cells. A graphical representation of the CyTOF data regarding our COVID-19 patients is reported in the Maxpar® Pathsetter™ Cen-se′™ plot (Fig. 6).

### Multiplex cytokine analysis

**Fig. 7:**
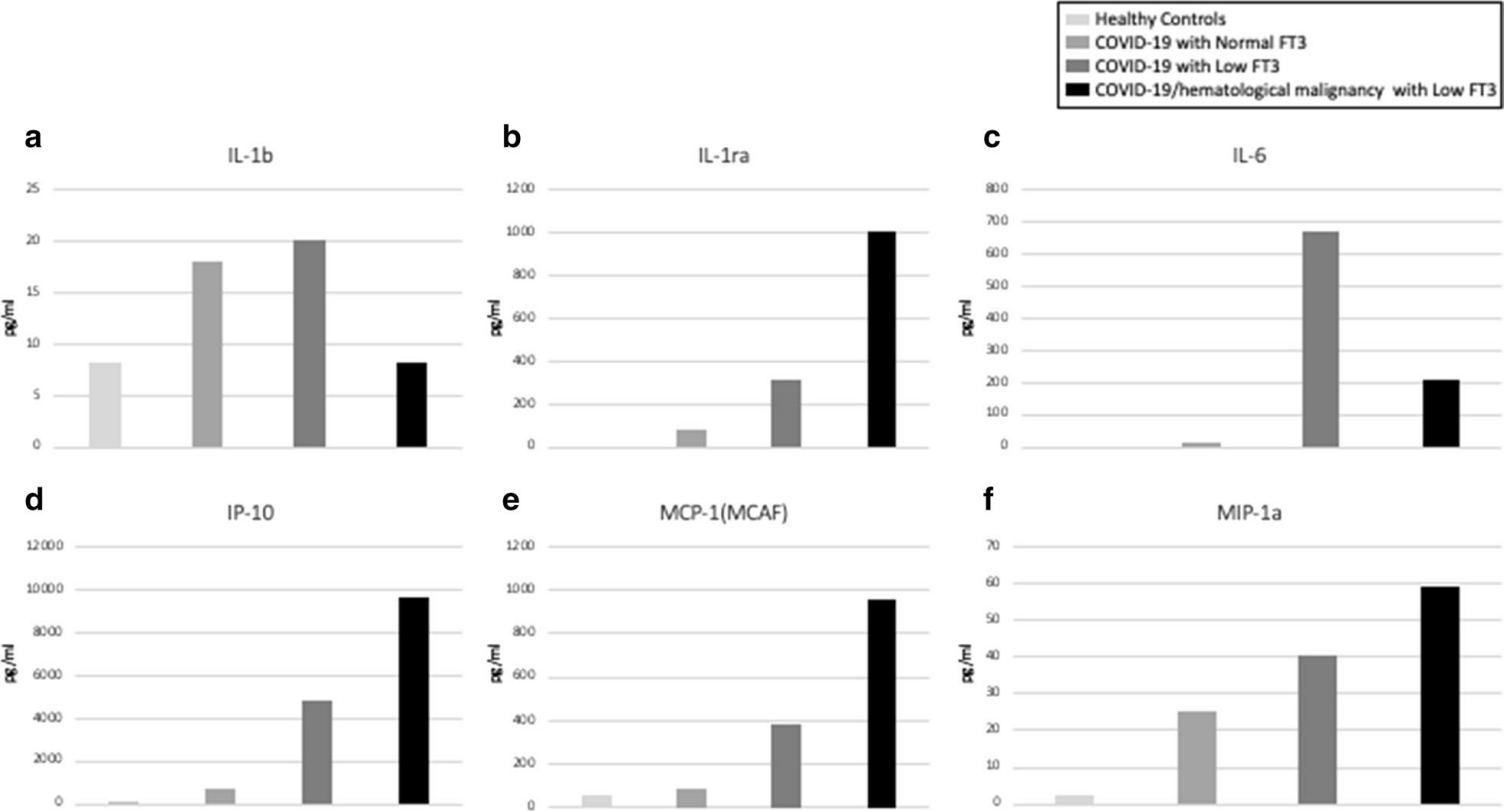
Multiplex cytokine analysis. Comparison of the mean serum levels of the cytokines, expressed in pg/ml, form healthy controls (light gray bars), from healthy controls (light gray bars), COVID-19 patients with normal FT3 serum levels (gray bars), from COVID-19 patients with low FT3 serum levels (dark gray bars) and from COVID-19 patients with hematological malignancy and with low FT3 levels (black bars). **a** IL-1b = interleukin 1 beta; **b** IL-1ra = interleukin-1 receptor antagonist; **c** IL-6 = interleukin 6; **d** IP-10 = interferon gamma-induced protein 10, also known as CXCL10; **e** MCP-1 = monocyte chemoattractant protein-1, also known as CCL2; **f** MIP-1a = macrophage inflammatory proteins-1a

Circulating cytokine analysis indicates that COVID-19 patients show an increase in many different cytokines and that their levels are influenced in COVID-19 patients by the occurrence of the low T3 syndrome (Fig. 7). We found a marked increase in the level of IL-6 in patients with low FT3 serum value, compared to those with normal FT3 serum levels (Fig. 7c). This result correlates with IL-6 classical laboratory immunological dosage (Table 3). It is relevant to note that the effect of low FT3 serum values on some cytokine levels is increased when hematological malignancy coexists with COVID-19. In the two patients with COVID-19 and hematological malignancy, in fact, there is an additional increase in the levels of IL-1ra, IP-10, MCP-1 and MIP-1a (Fig. 7b, d–f). The increase of these cytokines, observed in these patients, indicates that they may represent new targets of TH action, involved in the immune response to both COVID-19 and hematological malignancy.

### NanoString analysis

**Fig. 8:**
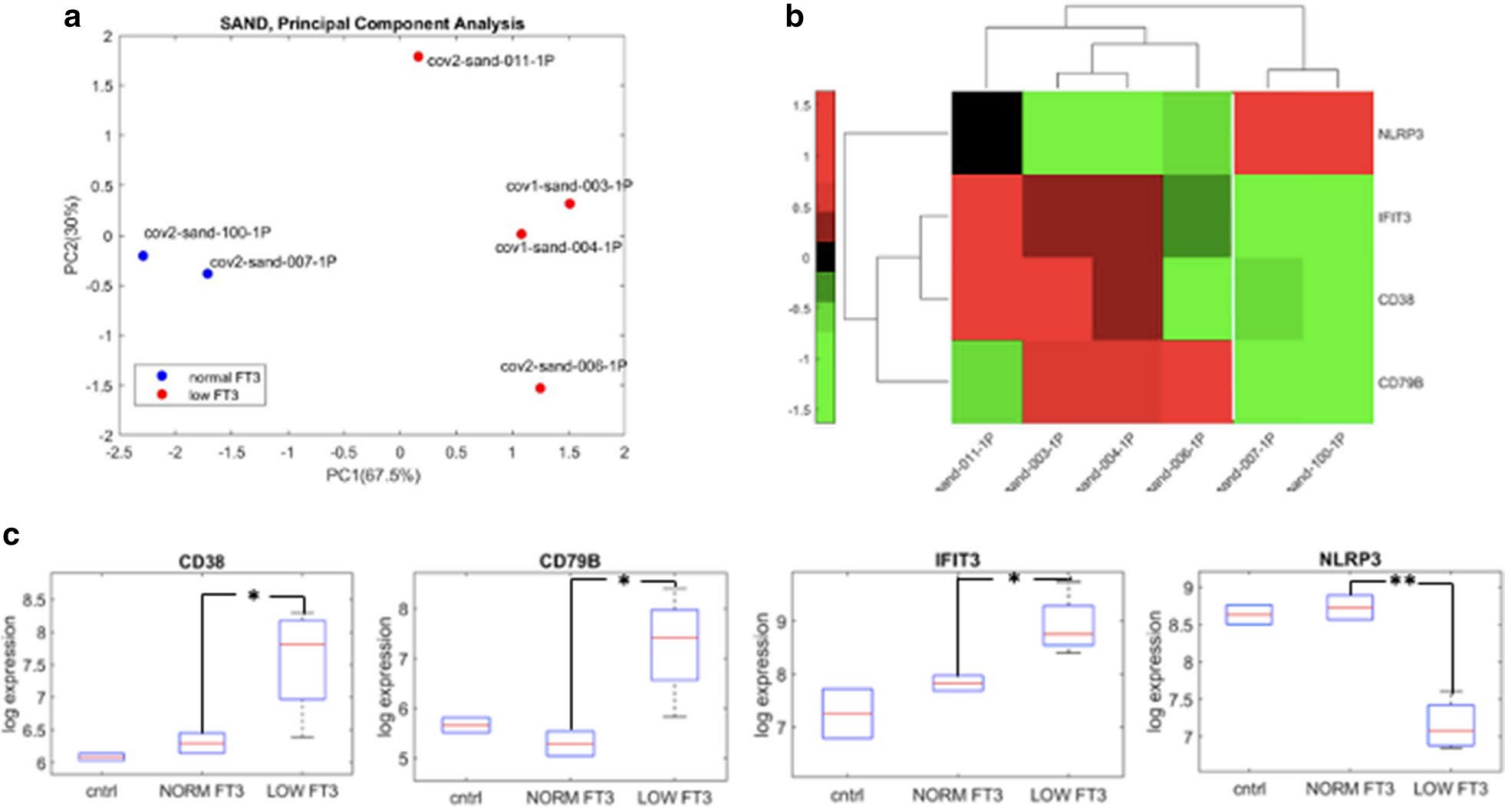
Gene signature analysis on our COVID-19 patients/low FT3 and COVID-19 patients/normal FT3. **a** Principal component analysis (PCA) applied to COVID-19 patients with low FT3 values (red circle) and those with normal FT3 values (blue circle) based on 4-gene signature; **b** unsupervised hierarchical clustered heatmap analysis for the Pearson correlation of absolute mRNA transcript abundance, as determined by NanoString. **c** Box-plot of log10(norm.counts) of the four genes contigs depending on FT3 serum levels, assessed by NanoString. The gene signature shows a different profile between COVID-19 patients in the presence or in the absence of the Low T3 syndrome. In particular, 3 genes are upregulated and 1 gene is downregulated in COVID-19 patients with low FT3 serum values, compared to patients with normal FT3 serum values. Patients Sand-006 and Sand-011 had an associated hematological malignancy. * means *p* value ≤ 0.05; ** means *p* value ≤ 0.005

Single cell based NanoString analysis indicates that COVID-19 patients with low FT3 serum values and those with normal FT3 values are clearly differentiated in the principal component analysis (PCA), with a different location in the plot. COVID-19 patients with normal FT3 serum values are located in the left side, while those with low FT3 serum values are located in the right sides respectively (Fig. 8a). Analysis of the genes that are mostly deregulated indicates that three genes are significantly upregulated and one is downregulated in patients with low serum FT3 levels, compared to those with normal FT3 ones (Fig. 8b). The upregulated genes are the CD38, the IFIT3 and the CD78B, while the downregulated is the NLRP3. In this latter case there is a highly statistically significant difference between the two groups of patients. The differences in the expression levels of these four genes in COVID-19 patients with low or normal FT3 serum values, compared to our control group of 2 non-COVID-19 patients, are reported in the box-plot analysis (Fig. 8c). The expression levels of these genes in the group of non-COVID-19 patients are similar to those observed in the group of COVID-19 patients with normal FT3 levels, but they clearly differ from those observed in the COVID-19 group of patients with low FT3 serum values. A more pronounced deregulation in these genes is observed when COVID-19 is associated with hematological malignancies. In particular, in the patient with COVID-19 and HD the expressions of the CD38 and of the IFIT3 genes are strongly increased, while in the patient with COVID-19 and CLL they are both normally expressed at a level comparable to that observed in the COVID-19 patients without the Low T3 syndrome. On the other hand, the patient with COVID-19 and HD doesn’t show the reduction in the expression level of the CD78B gene, seen in the other COVID-19 patients with the Low T3 Syndrome, including the patient with CLL, and the expression level of this gene is unchanged. Such results indicate that a specific pattern of gene expression is associated with THs action in COVID-19 with coexistent hematological malignancies. The down-regulation of CD79B expression appears to be specifically involved in HD, while the downregulation of both CD38 and IFIT3 appears to be specifically involved in the CLL.

## Discussion

The euthyroid sick syndrome (ESS) is a “hypothyroidic” condition that occurs in the absence of overt thyroid disease [25], reported many years ago in patients with systemic nonthyroidal illnesses (NTIs) [26–28], particularly those admitted to intensive care units [29]. These patients frequently present a fall in serum T3 levels that may be accompanied by a drop in the serum T4 levels. Serum TSH is usually inappropriately normal or slightly increased, but it may be also decreased. A reduced level of serum total T3 is rather frequent among hospitalized patients [30]. It is generally accepted that it represents an adaptive state of thyroid hormone (TH) economy. It has been associated with the severity of the case [31] and is considered a strong prognostic predictor of death in patients with heart disease [32]. A low serum T3 level predicts increased mortality also in patients with liver cirrhosis, advanced congestive heart failure [33, 34] and it is a strong predictor of poor prognosis in several other systemic illnesses, including surgery, multiple trauma, respiratory failure, septic shock, cerebrovascular diseases, cardiovascular diseases and severe burns [35–40]. Low T3 Syndrome was found to be a strong predictor of poor outcomes also in patients with community-acquired pneumonia [41] and in COVID-19 patients too [6]. Low T3 syndrome is also associated with metastasized cancer in hospitalized older population [42] and it is considered a poor prognostic factor for lung [43] and breast cancers [44]. The clinical and prognostic relevance of the Low T3 syndrome was reported also in different lymphoproliferative diseases [7–9].

THs play essential roles in both the innate and adaptive immune responses [45]. On the other hand, many proinflammatory cytokines could indirectly influence TH synthesis by lowering the serum TSH levels [46–48]. In this regard, a strong correlation has been previously reported between IL-6 and thyroid function and, in particular with the ESS [49]. IL-6 is elevated in the serum of COVID-19 patients, especially in those presenting with severe disease, as part of a cytokine storms [50]. In agreement with previous observations, we found increased levels of IL-6 in almost all patients. Patients belonging to Group A show higher values compared to those in Group B, but there is high variability among them and the difference is not statistically significant. In agreement, multiplex cytokine analysis indicates that IL-6 is highly increased in COVID-19 patients with low FT3 serum values (Fig. 7). In these experiments there is also a marked increase in the levels of IL-1ra, IP-10, MCP-1 and MIP-1a in patients with low FT3 serum values, compared to patients with normal FT3 serum values. The increase is much higher in patients with low FT3 and COVID-19 associated hematological malignancy. This observation suggests that such cytokine may be new targets of TH action on the immune cells and may play a role in the immune response to COVID and to hematological malignancy.

In all our patients there is a slight reduction in the absolute number of RBC and in the hemoglobin (Table 3). Such reduction is more evident in patients with low FT3 levels compared to those with normal levels of FT3 values. This finding is in agreement with the reported effect of THs on erythrocyte maturation [51]. However, the FT3 serum values mostly correlate with total WBC and lymphocyte subpopulations. An elevated ANC predicts ongoing inflammation and a decreased ALC is considered to be an indicator of poor prognosis, so a combination of these two measures, namely the NLR, is generally accepted to be predictive of an inflammatory situation [52] and of a more severe clinical condition of COVID-19 patients as well [53]. In our COVID-19 patients, an increased NLR correlates with the low FT3 serum levels. Such correlation appears much stronger when we consider the lymphocyte subpopulations and, more specifically, the T helper-inducer cells. Currently, limited information is available on the changes in lymphocyte subpopulations in patients affected by COVID-19 [54]. A significantly decreased number of T cells is considered a severity marker of the disease [55]. Both helper-inducer T cells and suppressor-cytotoxic T cells are reported to be below normal levels. However, lower levels of helper-inducer T cells are observed in the more severe group of patients. This is in agreement with our observations. We observed a more pronounced reduction of helper-inducer T cells in Group A patients, which are also characterized by higher SOFA and TSS scores, indicating a more severe disease. Single cells-based CytTOF analysis, performed in our small group of patients, confirms the reduction in the CD8+ and in the CD4+ T lymphocyte subpopulations, observed by FCA in COVID-19 patients with low FT3. Mass cytometry indicates that there is an increase in the CD4+ T lymphocytes in COVID-19 and associated hematological malignancy, due to an increase in the naïve subpopulation. The NK cells are increased too, with a relevant increase of both early and late types and this result is different from that observed by FCA, where the NK cells were reduced in patients with low FT3 serum levels. Mass cytometry has essentially the same workflow as conventional FCA and it has been recently compared to FCA and validated for its application in human oncology clinical trials [56].

Considering all these data, we can deduce that reduced FT3 levels can be considered as a severity disease biomarker of COVID-19. Our results are in agreement with those recently reported indicating reduced levels of FT3 serum levels as a prognostic tool for disease severity in the early presentation of COVID-19 [57]. It is interesting to observe that COVID-19 may even be responsible for the occurrence of a subacute thyroiditis, as recently reported in two reports [58, 59]. The potential use of treatment with intravenous high dose of T3 is under investigation in a new phase II randomized, double blind, placebo controlled trial (Thy-Support,ClinicalTrials.gov Identifier: NCT04348513) for enhancing recovery of critically ill COVID-19 patients [60]. The mechanism that is behind the T3 reduction in critical illness, as well in malignancies, is still under debate. Our study indicates that there are some possible targets of T3 action that are deregulated in COVID-19 patients showing the typical laboratory asset of the Low T3 syndrome, compared to COVID-19 patients that had normal FT3 serum values. We observed a different gene expression levels in the two patients that, in addition to the COVID-19, had hematological malignancies. Low T3 syndrome appears to be correlated not only with severity of COVID-19 but it is involved also in the mechanism of lymphoproliferative disorders that coexist with COVID-19. Thanks to NanoString analysis we identified a limited set of genes, all involved in immune reaction and expressed in immune cells, that may represent new potential targets of TH action on the immune system. The CD38, also known as cyclic ADP ribose hydrolase, is a glycoprotein that is present on the surface of many immune cells, including the CD4+, the CD8+, the B lymphocytes and the natural killer cells. It can function either as receptor or as an enzyme [61]. It is a well-recognized marker in leukemias, in myelomas and in different solid tumors [62–64]. CD 38 is a therapeutic target for many different hematological malignancies and there is a comprehensive list of monoclonal antibodies directed against the CD38 have been produced and approved. They have been designed to target the CD38, such as the daratumumab (Darzalex, Janssen Biotech, Inc. Horsham, PA), the daratumumab/hyaluronidase (Darzalex Faspro, Janssen Biotech, Inc. Horsham, PA), and the isatuximab (Sarclisa, Sanofi-Aventis, Bridgewater, NJ), or they are CD30-directed Antibody Drug conjugate (ADC), meaning that they consist of a targeted therapy monoclonal antibody and an antineoplastic (chemotherapy) agent that work together to destroy cancer cells, such as the brentuximab vedotin (Adcetris, Seattle Genetics, Inc. Bothell, WA). We detected an increased CD38 expression level in our patient affected by COVID-19 and a coexistent HD but not in the other patient that was affected by COVID-19 and a coexistent indolent CLL. The IFIT3 (Interferon Induced Protein With Tetratricopeptide Repeats 3) is a IFN-induced antiviral protein which acts as an inhibitor of cellular as well as viral processes, cell migration, proliferation, signalling, and viral replication. IFIT3 was highly upregulated in a human thyroid follicle culture system upon stimulation with a chemical analog of viral double-stranded RNA (dsRNA) to mimic viral infection [65]. CD79B, also known as B29 protein, is a component of the multimeric complex of the B lymphocyte antigen receptor [66]. It is expressed in the lymphocytes of patients with more advanced stages of CLL [67]. It represents a therapeutic target for the treatment of adult patients with relapsed or refractory diffuse large B-cell lymphoma and a specific monoclonal antibody has been product for this purpose. The Polatuzumab vedotin-piiq (POLIVY, Genentech, Inc. South San Francisco, CA) is an antibody–drug conjugate, composed of a monoclonal antibody against CD79B, in combination with bendamustine and a rituximab product. The NLRP3 is expressed predominantly on the macrophages and is considered a major component of the so-called inflammasome [68, 69]. NLRP3 plays a critical role for host immune defences against bacterial, fungal, and viral infections. NLRP3-induced pyroptosis and IL-1β/18 secretion is linked to various diseases. There are various inhibitors that specifically target the NLRP3 inflammasome [70]. We found that these genes are differentially regulated in COVID-19 patients in relation to the occurrence of the Low T3 Syndrome and to the presence of a hematological malignancy. Our study underlines the possible correlation among the immune response to viral infection, the thyroid function and the hematological malignancy and identifies these genes as new potential diagnostic prognostic and therapeutic targets for both COVID-19 and lymphoproliferative disorders. None of these genes has been previously reported to be regulated by THs. Reduced levels of FT3 in our COVID-19 patients affect differently the expression levels of these genes in the two lymphoproliferative diseases, suggesting that they act in a specific way and they depend on the type of the malignancy and possible on its stage. The major advantage of our study relies on the use of highly accurate methods, performed at the single cell level, to investigate the immunoprofiling and the gene expression patterns of COVID-19 patients with and without reduced FT3 serum levels. The major limitation is related to in the small number of samples that we have analyzed with these methods. Therefore, our observation needs to be confirmed in a larger population of COVID-19 affected patients.

## Conclusions

Our data, indicate that thyroid function alterations are frequently observed in the acute phase of COVID-19 disease. In these patients, low FT3 serum values appear to be associated with the severity serological indexes of the disease and, in particular, with an increase in the serum levels of markers of coagulation, inflammation and tissue damages. The strongest correlation is observed with increased NLR and dNLR ratios and with reduced level of T lymphocytes, especially of the helper-inducer T cell subpopulations. The low FT3 values are also associated with the clinical and radiologic scores of disease severity and can be considered as a prognostic marker of COVID-19. According to the results of our study, we should add the thyroid to the other endocrine glands, known to influence COVID-19 outcome, as reported by the European Society of Endocrinology [71]. FCA, CyTOF and multiplex cytokine analyses indicate that specific subset of circulating immune cells and cytokines are modulated by the T3 in COVID-19 patients and, finally, NanoString analysis allows us to identify four genes, responsive to THs, that may represent new diagnostic, prognostic and therapeutic targets in both COVID-19 and hematological malignancies.

## Supplementary Information

**Additional file 1: Figure S1.** **a** Graphic correlations between FT4 and TSH with serum FT3 values. **b** Box plot correlations between FT4 and TSH in COVID-19 patients with low serum FT3 values (Group A), compared to normal FT3 values (Group B). The corresponding correlation coefficient *r* for each scatter plot and the *p* value for each box plot are indicated.

**Additional file 2: Figure S2.** **a** Graphic and **b** box plot correlations between FT3 serum values and several disease severity markers in COVID-19 patients. The corresponding correlation coefficient *r* for each scatter plot and the *p* value for each box plot are indicated.

**Additional file 3: Figure S3.** **a** Correlations of FT3 serum values in COVID-19 patients with blood cells and disease markers; **b** correlations of FT3 serum values in COVID-19 patients with lymphocyte subpopulations.

## Data Availability

The datasets used and/or analyzed during the current study are available from the corresponding author on reasonable request. All data generated or analyzed during this study are included in this published article and its Additional files.

## Abbreviations

COVID-19: Coronavirus disease 2019
HD: Hodgkin disease
CLL: Chronic lymphocytic leukemia
MCcHL: Mixed Cellularity classical Hodgkin Lymphoma
SOFA: Sequential Organ Failure Assessment score
LIPI: Lung Inflammation Prognostic Index
TSS: Total Severity Score
CyTOF: Cytometry by Time-of-Flight
rRT-PCR: Real-time reverse-transcription polymerase chain reaction
FCA: Flow Cytometric Analysis
PCT: Procalcitonin
hs-CRP: High-sensitive C Reactive Protein
PT: Prothrombin time
LDH: Lactate DeHydrogenase
CK: Creatine Kinase
CK-MB: Creatine Kinase-Myocardial Band
hs-cTnI: Hypersensitive troponin I
IL-6: Interleukin-6
NLR: Neutrophil-to-lymphocyte ratio
dNLR: Derived neutrophil-to-lymphocyte ratio
PLR: Platelet-to-lymphocyte ratio
CT: Computerized tomography
PBMC: Peripheral blood mononuclear cells
HC: Healthy controls
Cen-se′: Cauchy Enhanced Nearest-neighbor Stochastic Embedding
PCA: Principal component analysis
COPD: Chronic obstructive pulmonary disease
RBC: Red blood cells
WBC: White blood cell
PLT: Platelets
ANC: Absolute Neutrophil Count
ALC: Absolute Lymphocyte Count
APC: Absolute Platelet Count
TH: Thyroid hormone
