# Supplementary figures and images for: Gene signature and immune cell profiling by high-dimensional, single-cell analysis in COVID-19 patients, presenting Low T3 syndrome and coexistent hematological malignancies
Source: J Transl Med. 2021 Apr 1;19:139. doi: 10.1186/s12967-021-02805-6 (PMC8016508; doi:10.1186/s12967-021-02805-6)

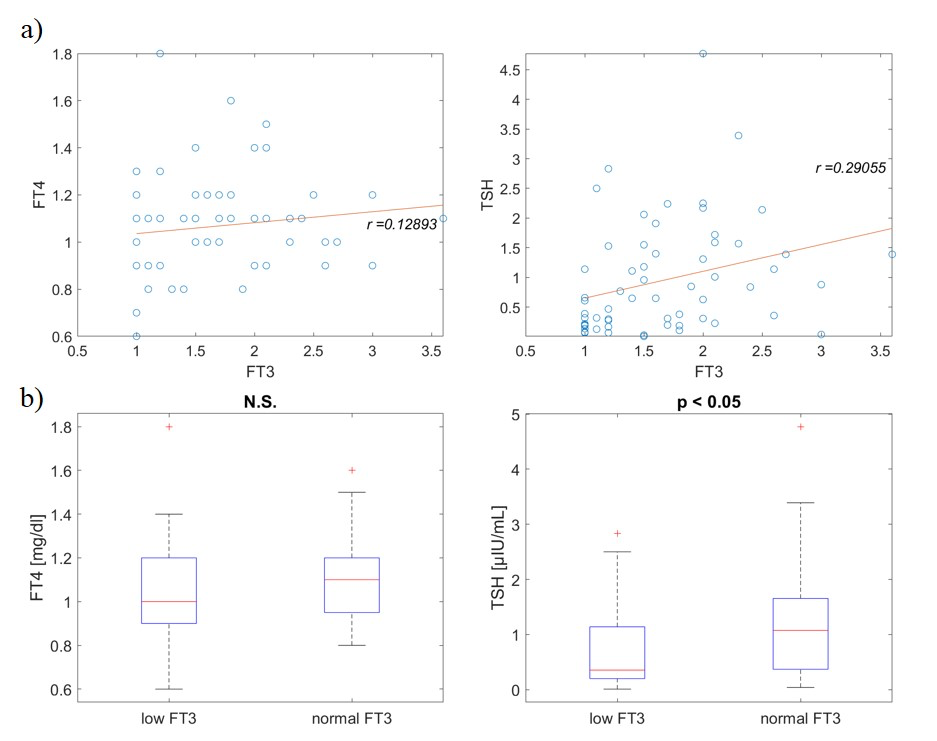

Supplement: Supplementary file 1 — Additional file 1: Figure S1. a Graphic correlations between FT4 and TSH with serum FT3 values. b Box plot correlations between FT4 and TSH in COVID-19 patients with low serum FT3 values (Group A), compared to normal FT3 values (Group B). The corresponding correlation coefficient r for each scatter plot and the p value for each box plot are indicated. [file 12967_2021_2805_MOESM1_ESM.tiff]

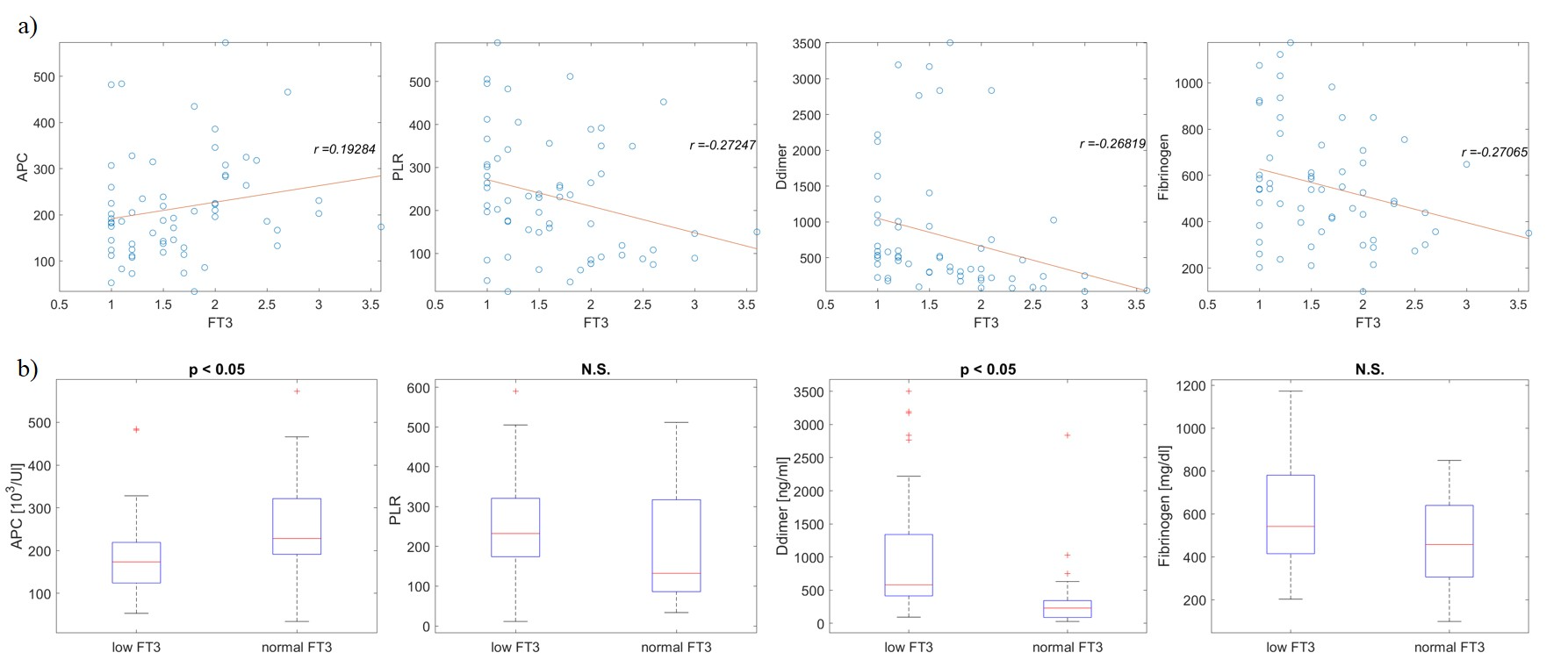

Supplement: Supplementary file 2 — Additional file 2: Figure S2. a Graphic and b box plot correlations between FT3 serum values and several disease severity markers in COVID-19 patients. The corresponding correlation coefficient r for each scatter plot and the p value for each box plot are indicated. [file 12967_2021_2805_MOESM2_ESM.tiff]

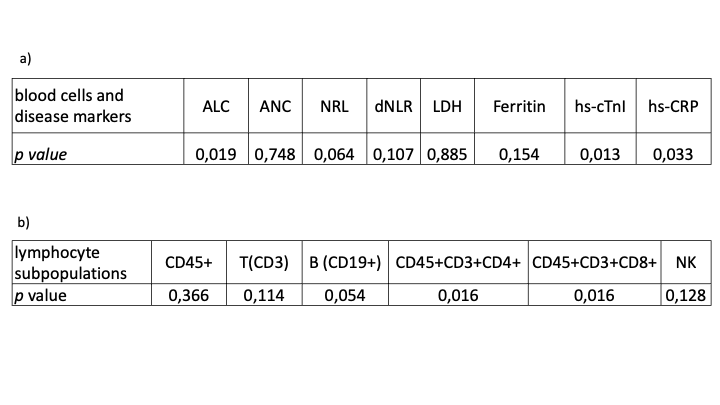

Supplement: Supplementary file 3 — Additional file 3: Figure S3. a Correlations of FT3 serum values in COVID-19 patients with blood cells and disease markers; b correlations of FT3 serum values in COVID-19 patients with lymphocyte subpopulations. [file 12967_2021_2805_MOESM3_ESM.tiff]
